# Supplementary figures and images for: Genetic diversity of enterotoxigenic Bacillus cereus strains in coriander in southwestern Mexico
Source: PeerJ. 2022 Jul 1;10:e13667. doi: 10.7717/peerj.13667 (PMC9252179; doi:10.7717/peerj.13667)

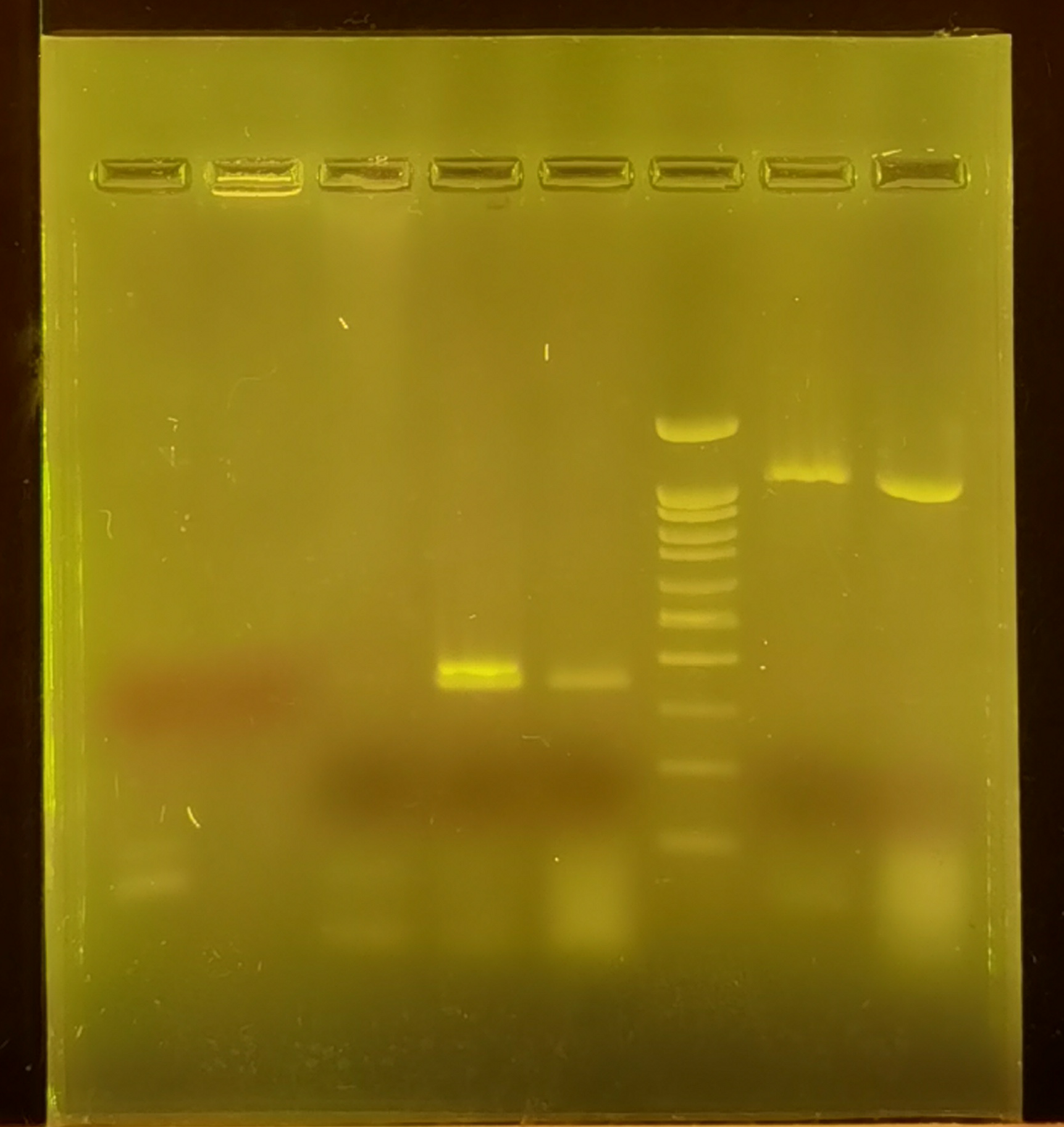

Supplement: Supplemental Information 2 — The GTG figure was taken in a different capture system [file peerj-10-13667-s002.zip › PNG/cytk hbl.png]

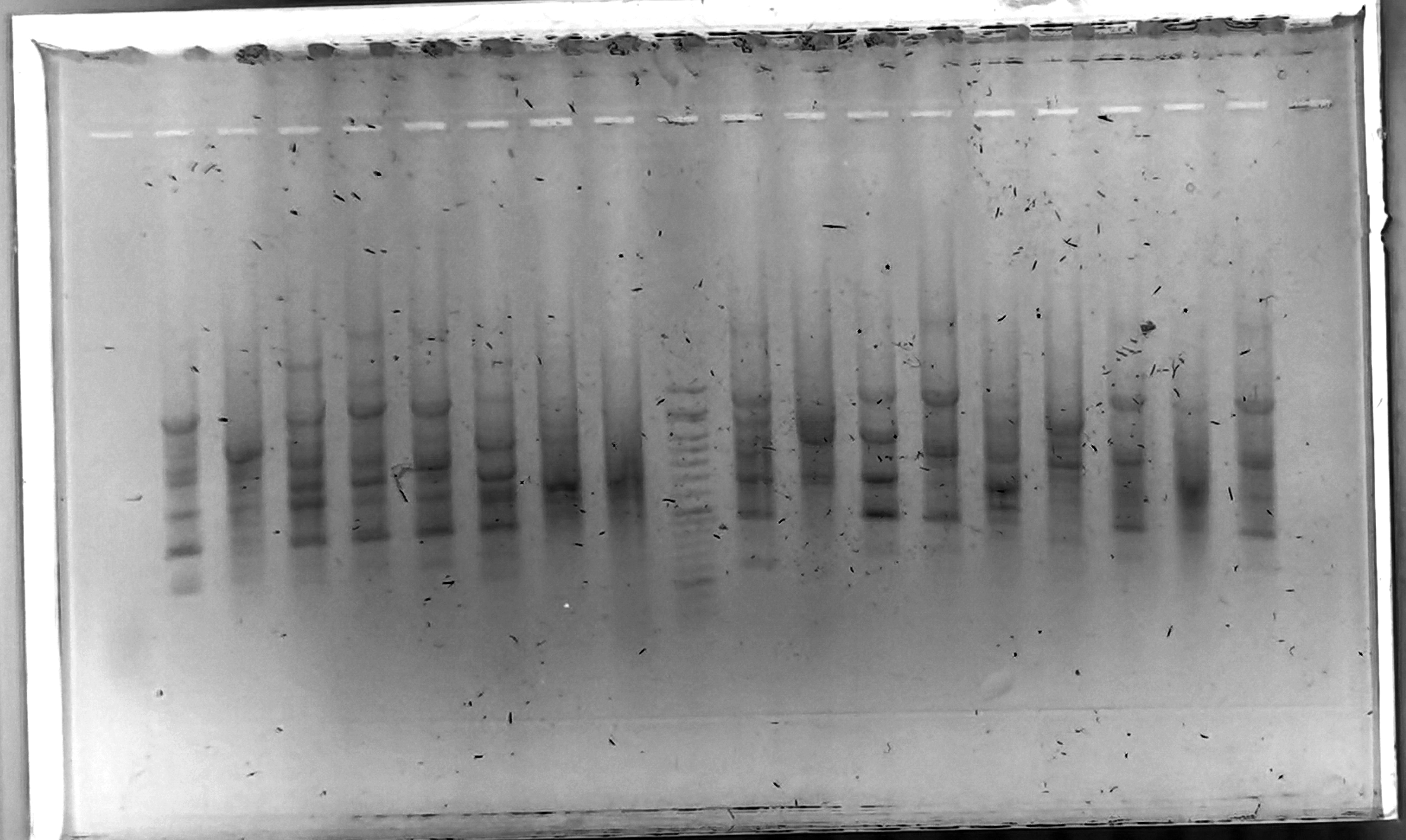

Supplement: Supplemental Information 2 — The GTG figure was taken in a different capture system [file peerj-10-13667-s002.zip › PNG/gtg Fig 3.png]

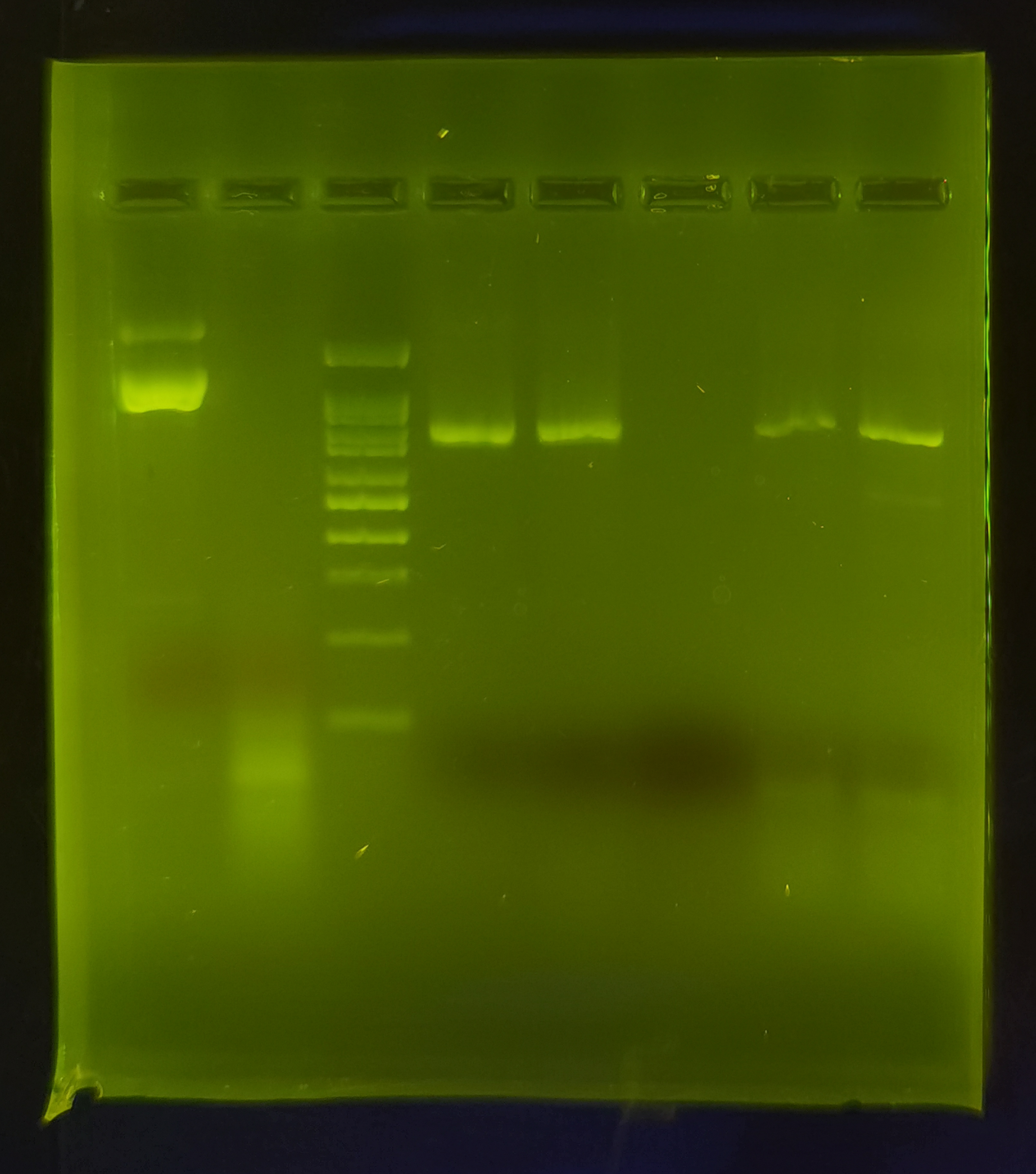

Supplement: Supplemental Information 2 — The GTG figure was taken in a different capture system [file peerj-10-13667-s002.zip › PNG/nhe Fig 1.png]

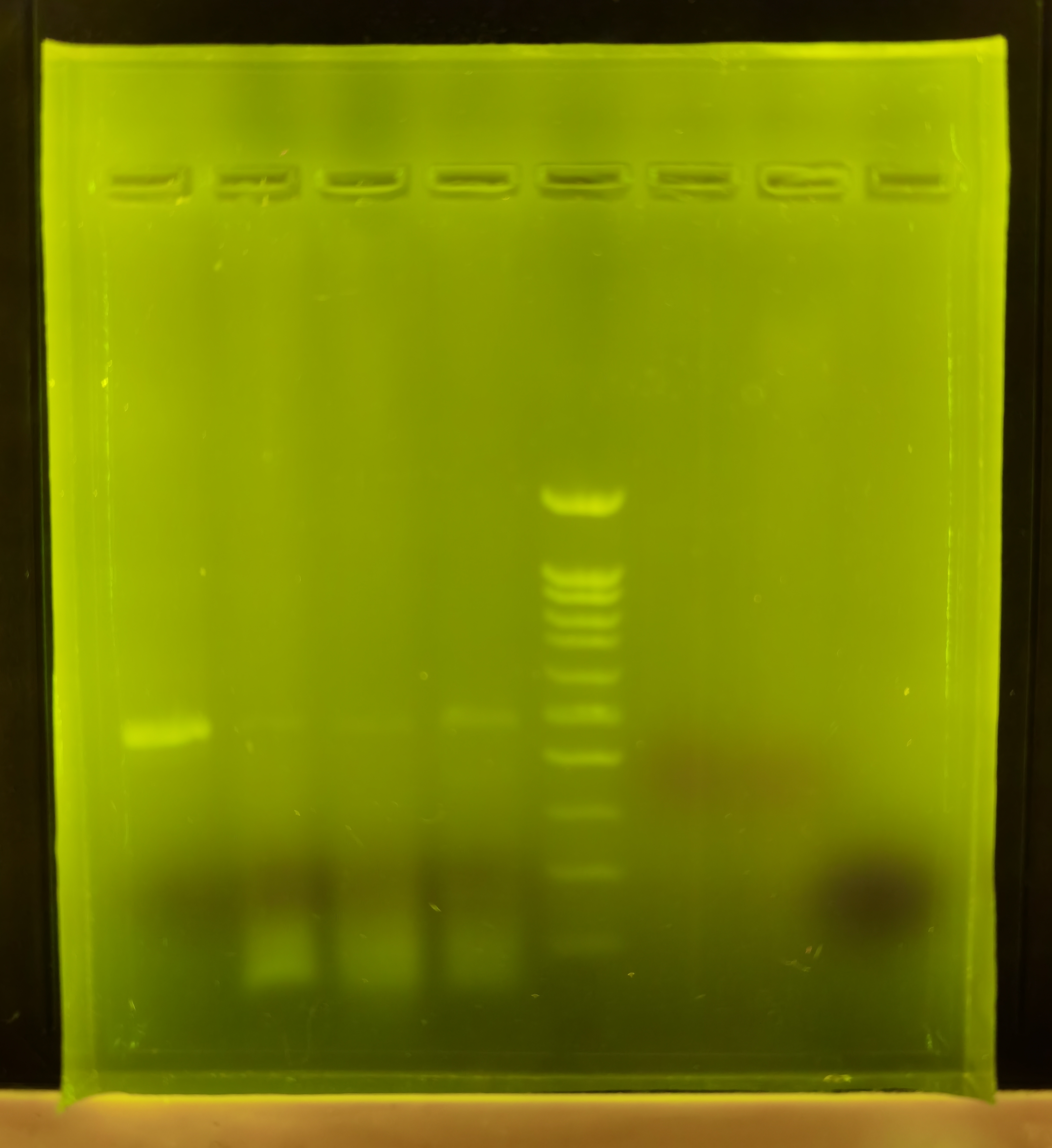

Supplement: Supplemental Information 2 — The GTG figure was taken in a different capture system [file peerj-10-13667-s002.zip › PNG/sipwtasA fig 2.png]
